# Effects of Different Substrates on Growth, Serum Biochemical Parameters, and Behavioral Characteristics of Juvenile Asian Giant Softshell Turtles, *Pelochelys cantorii*

**DOI:** 10.3390/ani16030383

**Published:** 2026-01-26

**Authors:** Xiangzhe Jia, Kai Cai, Liangyu Pan, Chengqing Wei, Wei Li, Xiaoli Liu, Xinping Zhu, Linmei Ye, Xiaoyou Hong

**Affiliations:** 1 College of Fisheries, Zhejiang Ocean University, Zhoushan 316022, China; 17861560201@163.com (X.J.); zhuxinping_1964@163.com (X.Z.); 2 Key Laboratory of Tropical & Subtropical Fishery Resource Application & Cultivation of Ministry of Agriculture, Pearl River Fisheries Research Institute, Chinese Academy of Fishery Sciences, Guangzhou 510380, China; 13868312246@163.com (L.P.); wcq1970@163.com (C.W.); liwei@prfri.ac.cn (W.L.); liu_xiaoli1988@126.com (X.L.); 3 Forestry Technology Extension Station, Qingtian County Forestry Bureau, Lishui 323900, China; lxck@stu.zafu.edu.cn; 4 State Key Laboratory for Development and Utilization of Forest Food Resources, Zhejiang A&F University, Hangzhou 311300, China

**Keywords:** Asian giant softshell turtle, behavioral characteristics, environmental adaptation, growth performance, serum biochemistry, substrate

## Abstract

The critically endangered Asian giant softshell turtle (*Pelochelys cantorii*) is a national first-class protected aquatic animal in China, and artificial breeding is vital for its conservation. Given the pivotal role of substrate in captive rearing, this study aimed to investigate the effects of different substrate types on the growth, serum biochemistry, and behavior of juvenile *P. cantorii*. A total of 45 8-month-old juveniles [(121.11 ± 0.65) g] were randomly allocated to three groups (fine sand [FS], pea gravel [PG], and no substrate [NS]) for an 18-day rearing trial. Results indicated that the FS and PG groups exhibited significantly higher weight gain and specific growth rates than the NS group (*p* < 0.01). Serum malondialdehyde (MDA) levels were lower in the FS and PG groups than in the NS group (*p* < 0.05), with no significant difference between FS and PG. Notably, three individuals in the NS group exhibited symptoms of skin ulceration. No significant intergroup differences were observed in glucose (GLU), triglyceride (TG), catalase (CAT), superoxide dismutase (SOD), glutathione peroxidase (GSH-Px), or cortisol (CORT) levels (*p* > 0.05). Behaviorally, the FS group demonstrated the highest hidden rest frequency and duration (*p* < 0.01) and significantly lower active avoidance behavior compared to PG and NS (*p* < 0.01). In conclusion, substrate type significantly influences captive juvenile *P. cantorii*, with fine sand being optimal as it enhances growth, alleviates oxidative stress, and reduces maladaptive behaviors.

## Simple Summary

The Asian giant softshell turtle (*Pelochelys cantorii*) is a highly protected aquatic animal on the brink of extinction, so artificial breeding is crucial for its survival. We aim to find out how different substrates in rearing containers—fine sand, pea gravel, or no substrate at all—affect juvenile turtles’ growth, serum biochemistry (the chemical makeup of their blood), and behavior. We raised 45 juvenile turtles for 18 days, splitting them evenly into three groups with the different bottom materials. Results showed young turtles in the fine sand and pea gravel groups grew faster than those with no substrate, and their blood had less malondialdehyde (MDA). Juveniles in the fine sand group also spent more time resting quietly in a hidden state and tried to escape much less often. No major differences were found in other blood chemical indicators across the three groups. This finding helps people choose the best substrate for raising juvenile *P. cantorii*, supporting their healthy growth and aiding the conservation of this endangered species.

## 1. Introduction

The Asian giant softshell turtle (*Pelochelys cantorii*) is one of the largest aquatic turtles inhabiting China’s inland waters. In recent times, illegal trade and excessive hunting have led to the continuous degradation of its habitats, a gradual reduction in its distribution range, and a sharp decline in its population. In 1989, it was listed as a Class I nationally protected aquatic wildlife species [1]. In 2018, it was listed as Critically Endangered on the IUCN Red List of Threatened Species [2]. In 2003, it was included in Appendix II of the Convention on International Trade in Endangered Species of Wild Fauna and Flora (CITES) [3]. In 2014, China achieved its first successful artificial breeding of *P. cantorii*. After nearly a decade of technical breakthroughs, the Gaoming *P. cantorii* Breeding and Protection Basein Foshan, Guangdong Province, has established a stable juvenile cultivation system, successfully maintaining over 1000 juveniles aged 1 to 9 years [4]. Although breakthroughs have been made in the *P. cantorii* artificial breeding techniques, current juvenile rearing occurs in relatively monotonous captive environments. Mass mortality incidents still occur during the rearing process, and it remains unclear whether the current rearing conditions represent the optimal growth environment for juveniles. Artificial captive rearing conditions are unable to replicate the complex structures of natural habitats, and the diverse ecological behaviors shaped by species evolution in the wild—such as foraging, predator avoidance, and territorial exploration—cannot be fully expressed in captive environments. The lack of natural behavioral stimuli in captivity may disrupt the animals’ physiological and psychological homeostasis, ultimately inducing a series of maladaptive traits. To further ensure the survival rate of artificially bred individuals, it is necessary to investigate the environmental adaptability of juvenile turtles in artificial settings.

Substrate is a key component of aquaculture ecosystems: it supplies nutrients, stabilizes water quality, and creates microhabitats for cultured animals and microorganisms. Most studies on substrate preference have focused on crustaceans, bivalves, and benthic fishes. For example, sand maximizes weight gain and survival of juvenile Dabry’s sturgeon (*Acipenser dabryanus*) [5] and juvenile Chinese mitten crabs (*Eriocheir sinensis*) prefer to burrow in mud or medium sand, whereas megalopae select muddy substrates [6], and substrate type significantly alters feeding and aggressive behavior in Chinese white shrimp (*Fenneropenaeus chinensis*) [7]. Comparable data for chelonians—especially large benthic softshell turtles such as *P. cantorii*—are scarce. Newly hatched *P. cantorii* remain in shallow water but move into deeper areas as body mass increases [8], so they interact continuously with the benthos. Indoor barrels, however, often lack natural substrata, potentially eliciting aberrant or absent behaviors that can compromise stress physiology and growth. It is hypothesized that the presence of substrate exerts a significant effect on the growth, physiological status, and behavioral traits of juvenile *P*. *cantorii*. To verify this assumption, we evaluated growth, serum biochemistry, and behavior of juvenile *P. cantorii* reared without substrate, on fine sand, or on pea gravel to identify the optimal substrate for early life stages, improve hatchery protocols, and guide habitat restoration for future stock enhancement.

## 2. Materials and Methods

### 2.1. Animal Ethics

All procedures were approved by the Laboratory Animal Ethics Committee of the Pearl River Fisheries Research Institute (Approval No. LAEC-PRFRI-2025-05-02).

### 2.2. Experimental Materials

The experiment was conducted in the juvenile *P. cantorii* rearing greenhouse at the Gaoming *P. cantorii* breeding and protection base (Foshan, Guangdong Province, China). The experimental animals consisted of 8-month-old juvenile *P. cantorii* reared in circular tanks with a diameter of 240 cm. A total of 45 individuals were selected from 85 healthy 8-month-old juveniles in the tanks, with the selection criteria including the absence of external injuries and deformities, normal locomotor ability, and a uniform mean body weight of (121.11 ± 0.65) g.

### 2.3. Aquaculture Environment

**Figure 1:**
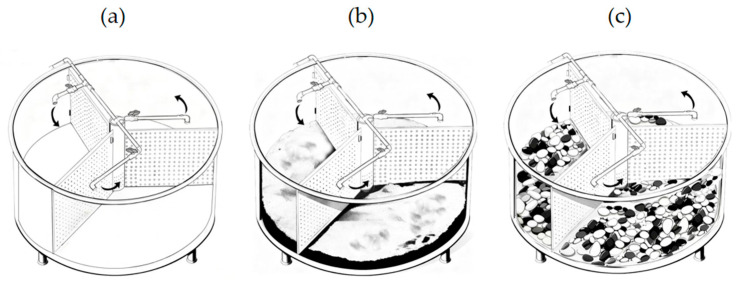
Schematic diagram of aquaculture experiment barrel: (**a**) no-substrate group; (**b**) fine sand group; and (**c**) pea gravel group.

Three circular barrels (diameter 120 cm, height 50 cm) were filled with 20 cm of filtered recirculating water (pH 7.0–7.5; NH_3_-N ≤ 0.5 mg/L; 26.0–27.5 °C). Each barrel received one substrate to a depth of 10 cm: no-substrate (NS), fine sand (FS, 0.5–1 mm), or pea gravel (PG, 10–20 mm). Barrels were partitioned into three compartments, yielding three replicates per treatment (Figure 1). The fine sand and pea gravel were subjected to a sterilization procedure prior to use: first soaked in 5% potassium permanganate solution for 20 min, then thoroughly rinsed with clean water, and finally air-dried under sunlight. Subsequently, these substrates were placed into the barrels alongside the no-substrate group, followed by a one-week water quality regulation period to stabilize the water conditions prior to the initiation of the formal experiment. *P. cantorii* were fed 2–4 cm mosquito fish (*Gambusia affinis*) daily at dusk; leftovers were removed and replaced every 48 h. After seven days of acclimation, the 18-day experiment began.

### 2.4. Experimental Design and Process

Forty-five juveniles were randomly assigned to three barrels, with five individuals placed in each of the nine partitioned zones (as shown in Figure 1). Night-vision cameras (DS-2CD1225D-I3, Hikvision, Hangzhou, China) were installed above each barrel for observation and recording. On the 18th day of the experiment, skin ulceration symptoms were observed on the carapaces and plastron skirts of three experimental individuals in the NS group. Consequently, the experiment was terminated in strict accordance with the guidelines of the Laboratory Animal Ethics Committee of the Pearl River Fisheries Research Institute. The affected individuals were isolated individually for treatment to avoid further suffering and ensure compliance with animal welfare standards. Following the experiment, final body weights were measured for all turtles. Blood samples (1 mL) were randomly collected from the jugular vein of one turtle per compartment for serum analysis. Serum concentrations of glucose (GLU), triglycerides (TGs), superoxide dismutase (SOD), catalase (CAT), glutathione peroxidase (GSH-PX), malondialdehyde (MDA), and corticosterone (CORT) were subsequently determined. GLU, TG, SOD, and CAT were measured using Wuhan Servicebio Kits (Wuhan Servicebio Technology Co., Ltd., Wuhan, China), while CORT was detected using Shanghai Jonlnbio ELISA Kits (Shanghai Jianglai Biotechnology Co., Ltd., Shanghai, China). Additionally, 24 h video recordings from the final day of the experiment were copied for behavioral analysis.

### 2.5. Computational and Statistical Analysis

#### 2.5.1. Growth Performance Indicators

Weight gain rate (WGR) and specific growth rate (SGR) were calculated separately according to the following formula:

WGR = (W_t_ − W_0_)/W_0_ × 100

SGR = (lnW_t_ − lnW_0_)/T × 100

In the equation, W_0_ and W_t_ represent the body weights of the juvenile turtles at the start and end of the experiment, and T was the duration of the experiment.

#### 2.5.2. Behavioral Characteristics

Using continuous focal sampling and total event sampling methods, and based on the behavioral spectrum classification criteria for juvenile *P. cantorii* [9], one juvenile turtle was randomly selected from each area as an observation subject. The occurrence frequency and duration of various behaviors in selected fixed individuals within a 24 h period were observed, and the frequency proportion and time proportion of each behavior were calculated. All behavioral observations in this study were conducted by a single systematically trained and calibrated observer. Prior to commencing formal behavioral observation and sampling, the observer first classified and documented behaviors from select surveillance footage across the three experimental groups. Following a one-week interval, the same footage was re-analyzed independently by the observer. Formal data collection was initiated only after consistent results were achieved between the two rounds of analysis.

All data were analyzed with SPSS 23.0 and are reported as mean ± SD. The Shapiro–Wilk test and Levene test were used to evaluate the normal distribution and variance homogeneity of experimental data, respectively. Differences among groups were tested by one-way ANOVA followed by Duncan post hoc comparisons. Figures were prepared in GraphPad Prism 8. Significance was set at *p* < 0.05 and high significance at *p* < 0.01.

## 3. Results

### 3.1. Effects of Different Substrates on the Growth Performance of Juvenile P. cantorii

Table 1 shows that juveniles on FS attained a weight-gain rate of (17.47 ± 0.58)% and a specific growth rate of (89.45 ± 3.83)%/day, whereas those on PG reached (16.09 ± 1.00)% and (82.90 ± 5.06)%/day, respectively; NS gained only (3.21 ± 1.73)% and grew at (17.68 ± 8.32)%/day. Values for the two substrate treatments were similar (*p* > 0.05) but were both significantly higher than those for the NS group (*p* < 0.01), indicating that the presence of substrate strongly promotes growth in juveniles.

### 3.2. Effects of Different Substrates on Serum Biochemical Indicators in Juvenile P. cantorii 

#### 3.2.1. Effects of Different Substrates on Metabolic and Energy Balance Indicators in Serum of Juvenile *P. cantorii*

**Figure 2:**
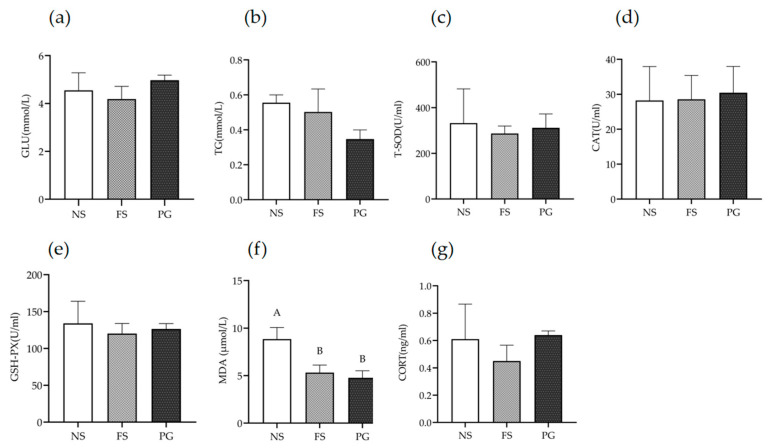
Effects of different substrates on serum biochemical indicators in juvenile *P. cantorii*. (Mean ± SD, N = 3). N = 3 indicates that the replicate units are barrels, with data analyzed based on the mean values of the three compartments per barrel. FS—fine sand; PG—pea gravel; NS—no substrate. (**a**) Serum Glu levels; (**b**) serum TG levels; (**c**) serum T-SOD levels; (**d**) serum CAT levels; (**e**) serum GSH-PX levels; (**f**) serum MDA levels; and (**g**) serum CORT levels. Different uppercase letters denote highly significant differences (*p* < 0.01).

Figure 2a shows serum glucose averages of (4.97 ± 0.21) mmol/L in the PG group, (4.55 ± 0.73) mmol/L in the NS group, and (4.19 ± 0.53) mmol/L in the FS group, with no significant differences (*p* > 0.05). Figure 2b reveals a similar pattern for triglycerides: 0.56 ± 0.45 mmol/L (NS), 0.50 ± 0.13 mmol/L (FS), and 0.35 ± 0.53 mmol/L (PG), again with no significant variation among treatments (*p* > 0.05).

#### 3.2.2. Effects of Different Substrates on Oxidative Stress Markers in Serum of Juvenile *P. cantorii*

Serum T-SOD activity was highest in the NS group, followed by PG and then FS, but the difference was not significant (*p* > 0.05, Figure 2c). CAT activity followed the same ranking—PG > NS > FS—with no significant variation among treatments (*p* > 0.05, Figure 2d). GSH-Px levels also declined from NS to PG to FS, yet remained statistically similar (*p* > 0.05, Figure 2e). In contrast, MDA concentration was markedly higher in the no-substrate group (8.85 ± 1.21 µmol/L) than in either substrate group (*p* < 0.05), while FS and PG values did not differ from each other (*p* > 0.05, Figure 2f).

#### 3.2.3. Effects of Different Substrates on Stress Hormone Levels in Juvenile *P. cantorii* Serum

Cortisol levels were highest in the PG group (0.64 ± 0.03 ng/mL), followed by the NS group (0.61 ± 0.26 ng/mL) and lowest in the FS group (0.45 ± 0.12 ng/mL), with no significant differences among treatments (*p* > 0.05, Figure 2g).

### 3.3. Effects of Different Substrates on Juvenile P. cantorii Behavioral Characteristics

Appendix A Table A1 and Table A2 show that behavior differed markedly among treatments. Juveniles on FS spent (93.51 ± 2.28)% of the time hidden, a value highly significantly greater than that recorded for the NS (0 ± 0) and PG [(51.75 ± 0.20)%] groups (*p* < 0.01); the latter two also did differ from each other (*p* < 0.01). Active avoidance behavior in the NS treatment reached (34.39 ± 5.32)% events and occupied (52.08 ± 14.84)% of the observation period, both significantly higher than in the PG and FS groups (*p* < 0.01). Digging and burying behavior exhibited a significantly higher frequency proportion for FS (27.51% ± 0.29%) compared to PG (17.16% ± 3.7%) and NS (0 ± 0); the time proportion for FS (0.46% ± 0.02%) showed a highly significant difference from PG (0.95% ± 0.31%) (*p* < 0.01). Moreover, both FS and PG groups demonstrated significantly higher proportions than NS (0 ± 0). The FS group’s turtles displayed the richest behavioral repertoire (5 categories, 16 acts), and surfaced at a regular 10 min interval.

## 4. Discussion

### 4.1. Effects of Different Substrate Types on Juvenile P. cantorii Growth

Aquaculture studies consistently show that the presence and type of substrate can strongly affect growth rate and weight gain. Scholars have already found that smooth softshell turtle (*Apalone mutica*) hatchlings maintained on a sand substrate grew faster and differed in body appearance compared to conspecific hatchlings maintained without a sand substrate [10]. Ponds containing substrate can enhance growth of school prawn (*Metapenaeus macleayi*) [11]. In the present trial, juveniles on either fine sand or pea gravel grew significantly faster than those in no-substrate group, a pattern that aligns with studies of other benthic species [7,12]. Both *M. macleayi* and *P. cantorii* rely heavily on substrate for routine activities, which may explain the similar growth response. Thus, substrate appears critical for benthic taxa; juveniles on fine sand could bury themselves completely, whereas those on pea gravel could still partially conceal their bodies. Weight gain and specific growth rate were slightly higher on sand than on gravel, but the difference was not statistically significant, presumably because the 18-day trial was too short for the divergence to accumulate.

### 4.2. Effects of Different Substrate Types on Serum Biochemical Parameters in Juvenile P. cantorii

Blood is a major component of the internal milieu and helps maintain homeostasis; serum chemistry values therefore reflect an animal’s physiological state, metabolic rate, and species-specific traits [13]. GLU is the primary circulating energy source, and its concentration indicates carbohydrate metabolism [14]. In this study, GLU was higher in the no-substrate and pea gravel groups than in the fine-sand group, but the difference was not statistically significant. The same trend has been reported for Chinese sturgeon (*Acipenser sinensis*) held under sub-optimal conditions [14]. TGs reflect lipid metabolism and energy storage [15]; the highest TG values in the no-substrate group may indicate that the absence of substrate could affect the lipid metabolism level of juvenile *P. cantorii*. CORT is the main glucocorticoid in reptiles and a standard indicator of stress [16,17]. Meanwhile, elevated corticosterone would enhance gluconeogenesis and raise blood glucose. In this study, CORT levels did not differ among treatments, yet values were numerically highest in the no-substrate and pea gravel groups and lowest in the fine-sand group. Numerical trends in GLU and CORT may suggest a mild stress response in NS and PG groups, though these were not statistically confirmed in this short-term study. This speculation is consistent with previous findings from relevant studies on zebrafish (*Danio rerio*) [18].

In healthy aerobic organisms, the production and elimination of reactive oxygen species (ROS) are balanced [19]. When this balance is disrupted, oxidative stress occurs. T-SOD is the first line of defense, converting superoxide radicals (O_2_^−^) into H_2_O_2_ [20,21], while GSH-Px acts as the second line by catalyzing the breakdown of H_2_O_2_ and lipid peroxides [22]. In this study, serum T-SOD and GSH-Px activities were highest in the NS group, followed by the PG and FS groups. During the early stage of stress, an aquatic organism adapts to the stress by increasing the activities of antioxidant enzymes and level of GSH [23]. The elevated activities of T-SOD and GSH-Px in the NS group may represent a compensatory upregulation of antioxidant defenses in response to increased ROS production caused by chronic stress and high activity levels. However, alternative explanations cannot be excluded, such as baseline metabolic differences among individual *P. cantorii*.

MDA, a final product of lipid peroxidation, reflects the degree of oxidative damage to cell membranes [24]. In this experiment, MDA levels were significantly higher in the NS group compared to the PG and FS groups, indicating more severe oxidative damage. This is likely due to the lack of shelter in the no-substrate environment, which increased the frequency and duration of stereotypic swimming and active avoidance behaviors. Excessive activity may have led to overproduction of free radicals, elevated lipid peroxidation, and a sharp rise in MDA as the body attempted to restore internal balance and mitigate oxidative damage [25,26]. Studies have shown that chronic exposure to thermal stress can reduce the concentrations of TGs and GSH-Px in the serum of juvenile Greater Amberjack (*Seriola dumerili*), decrease the activities of superoxide SOD and CAT, and increase the concentration of MDA. In studies on the silver scat (*Selenotoca multifasciata*), researchers also observed that under different environmental treatments, the differences in the activities of certain antioxidant enzymes were not statistically significant and only showed a tendency toward divergence, whereas significant differences were detected in other antioxidant indices [27]. This is partially consistent with the findings of the present study.

Previous studies have shown that oxidative stress not only correlates with external surface lesions but also disrupts immune homeostasis and function, rendering aquatic animals more vulnerable to infections [28]. This may explain the aggravated skin ulceration in three individuals from the no-substrate group, a hypothesis that requires further in-depth research.

The present study has certain limitations. Given the slow growth rate of *P*. *cantorii*, the 18-day experimental duration only reflects the acute responses of juveniles to substrate conditions, and thus fails to fully capture their long-term growth trends and dynamic changes in serum parameters.

### 4.3. Effects of Different Substrate Types on Juvenile P. cantorii Behavioral Characteristics

Numerous studies have noted that blood chemistry alone gives only a partial picture of stress in reptiles; behavior must be included for a reliable assessment [29]. Comparing behavioral profiles across groups and tracking how the same group reacts to habitat change are now standard tools in endangered-species management [30,31]. In this study the three treatments produced sharply different behavioral spectra. Juveniles in bare barrels and gravel barrels spent most of their time repeatedly attempting to escape through high-frequency swimming and scrambling to climb along the edges and corners, whereas those on fine sand remained quietly buried and surfaced at regular, predictable intervals.

Prolonged, fixed-pattern swimming along barrel walls is a classic stereotypy in captive aquatic animals [32,33]. When reptiles repeatedly bump, climb, or scrape the enclosure, or when activity exceeds that seen under natural conditions, the animal is usually signaling acute stress [29]. Without substrate, the hatchlings had no refuge, felt exposed, and responded by continual swimming and wall-clambering in search of cover or an exit. Meanwhile, during the process of climbing the barrel walls, multiple juveniles were highly prone to mutual stacking. Individuals could be scratched by the claws of their conspecifics in this process, which may account for the skin ulceration observed in some juveniles of the no-substrate group. Pea gravel offered some cover, but particle size and weight prevented the turtles from excavating a stable burrow; half-exposed, they still tried to flee.

Behavior on fine sand mirrored that of wild freshwater turtles. Field studies show that these species spend most of the day motionless, moving only to feed, avoid predators, reproduce, or migrate, and all movements follow an energy-saving strategy that avoids useless expenditure [34]. The sand grain-size matched the turtles’ ability to dig shallow pockets in which they lay buried except for the snout, replicating the sandy riverbeds wild *P. cantorii* prefer. This habitat compatibility reduced stress, produced a natural time budget by prolonged resting and regular surfacing for breathing, and yielded the richest behavioral repertoire. In short, fine sand recreated a natural refuge, satisfied the species’ innate burying drive, minimized abnormal behaviors, and preserved physiological homeostasis, whereas bare barrels and pea gravel—through poor habitat fit—elicited pronounced stress that compromised both growth and health.

## 5. Conclusions

This study—combining growth performance, serum biochemistry, and behavioral data—demonstrates that substrate type is a critical driver of environmental adaptation in juvenile *P. cantorii*. Fine sand, by mimicking natural riverbeds, curbs stress-related behaviors, stabilizes internal homeostasis, and supports normal physiology. In contrast, bare barrels or gravel fail to satisfy the turtles’ innate need to burrow and hide, triggering a pronounced stress response: activity soars, feeding drops, and excess energy expenditure lowers growth. These findings provide direct guidance for substrate selection in the captive rearing of *P*. *cantorii*, and lay a foundation for optimizing artificial breeding protocols. Meanwhile, they establish a reference framework for the acclimation and adaptation of endangered juveniles prior to their release into the wild, and offer a scientific basis for formulating conservation policies targeting the natural riverbed ecosystems of wild *P*. *cantorii* populations, thereby contributing to the global conservation efforts for critically endangered chelonian species.

## Figures and Tables

**Table 1:** Effects of different substrates on growth performance of juvenile *P. cantorii* (mean ± SD, N = 3).

Item	Group			*F*	DF (BG, WG)	*p* Values	η_P_^2^ (Effect Size)	95% CI (FS vs. NS)
	FS	PG	NS					
Initial weight	121.39 ± 2.39	120.36 ± 0.85	121.57 ± 3.67	0.19	2, 6	0.826	-	-
(g)								
Final weight	142.59 ± 2.71 ^A^	139.73 ± 1.40 ^A^	125.48 ± 2.23 ^B^	50.76	2, 6	<0.001	0.944	13.21–20.91
(g)								
Weight gain rate	17.47 ± 0.58 ^A^	16.09 ± 1.00 ^A^	3.21 ± 1.73 ^B^	84.93	2, 6	<0.001	0.965	11.53–17.01
(%)								
Specific growth rate	89.45 ± 3.83 ^A^	82.90 ± 5.06 ^A^	17.68 ± 8.32 ^B^	109.67	2, 6	<0.001	0.973	58.72–85.02
(%/d)								

Abbreviations: FS—fine sand; PG—pea gravel; NS—no substrate; DF—degree of freedom; BG—between group; and WG—within group. η_P_^2^ = partial eta squared (effect size: <0.01 = small, 0.01–0.06 = medium, and >0.06 = large); 95% CI = 95% confidence interval. 95% CI is presented for significant differences between FS (optimal substrate) and NS (control group); “-” indicates no significant difference (*p* > 0.05). Identical letters indicate no significant difference; different capital letters indicate a highly significant difference (*p* < 0.01). Note: In this table, N = 3 indicates that the replicate units are barrels, with data analyzed based on the mean values of the three compartments per barrel.

## Data Availability

All data generated and analyzed during this study are included in the published article.

## Appendix A

**Table A1:** Effects of different substrate types on the frequency of juvenile *P. cantorii* behaviors (mean ± SD, N = 3).

Behavior Type [9]	Substrate Type	Behavior Frequency	*F*	DF (BG, WG)	*p* Values	η_P_^2^ (Effect Size)	95% CI (FS vs. NS)
		(%)					
Bite food	FS	0.29 ± 0.36	-	-	-	-	-
	PG	0.32 ± 0.28	-	-	-	-	-
	NS	0.21 ± 0.36	-	-	-	-	-
Swallow food	FS	0.29 ± 0.36	-	-	-	-	-
	PG	0.32 ± 0.28	-	-	-	-	-
	NS	0.10 ± 0.18	-	-	-	-	-
Predation	FS	0.29 ± 0.36	-	-	-	-	-
	PG	0.32 ± 0.28	-	-	-	-	-
	NS	0.10 ± 0.18	-	-	-	-	-
Patrolling	FS	1.03 ± 0.61 ^a^	-	-	-	-	-
	PG	0 ^b^	16.84	2, 6	0.003	0.848	0.45–1.61
	NS	0 ^b^	-	-	-	-	-
Extended observation	FS	3.01 ± 1.41	-	-	-	-	-
	PG	3.92 ± 4.19	-	-	-	-	-
	NS	0	-	-	-	-	-
Roaming	FS	1.40 ± 0.59 ^A^	-	-	-	-	-
	PG	0 ^B^	22.94	2, 6	<0.001	0.885	0.78–2.02
	NS	0 ^B^	-	-	-	-	-
Climbing	FS	0.13 ± 0.23	-	-	-	-	-
	PG	0	-	-	-	-	-
	NS	0	-	-	-	-	-
Digging and burying *	FS	27.51 ± 0.29 ^A^	-	-	-	-	-
	PG	17.16 ± 3.7 ^B^	189.35	2, 6	<0.001	0.984	25.63–29.39
	NS	0 ^C^	-	-	-	-	-
Out of the water	FS	1.56 ± 0.85	-	-	-	-	-
	PG	0	-	-	-	-	-
	NS	0.78 ± 1.35	-	-	-	-	-
Slow crawling	FS	5.32 ± 2.30 ^B^	-	-	-	-	-
	PG	8.94 ± 0.55 ^A^	7.85	2, 6	0.024	0.722	−5.71–−0.41
	NS	8.38 ± 1.47 ^A^	-	-	-	-	-
Going down the barrel	FS	0.92 ± 0.30 ^B^	-	-	-	-	-
	PG	0 ^C^	64.1	2, 6	<0.001	0.957	−1.45–−0.35
	NS	1.82 ± 0.66 ^A^	-	-	-	-	-
Quick touring	FS	0.57 ± 0.40 ^a^	-	-	-	-	-
	PG	0 ^b^	5.55	2, 6	0.045	0.649	−0.73–0.59
	NS	0.64 ± 0.56 ^a^	-	-	-	-	-
Active avoidance *	FS	1.35 ± 1.34 ^C^	-	-	-	-	-
	PG	9.06 ± 0.82 ^B^	114.39	2, 6	<0.001	0.974	−37.61–−28.47
	NS	34.39 ± 5.32 ^A^	-	-	-	-	-
Hidden rest *	FS	27.51 ± 0.29 ^A^	-	-	-	-	-
	PG	12.75 ± 7.35 ^B^	24.62	2, 6	0.001	0.894	25.63–29.39
	NS	0 ^C^	-	-	-	-	-
Lying down	FS	1.95 ± 2.29 ^C^	-	-	-	-	-
	PG	24.89 ± 11.86 ^B^	18.51	2, 6	0.002	0.861	−36.09–−26.75
	NS	33.37 ± 1.96 ^A^	-	-	-	-	-
breathe	FS	26.54 ± 3.17	-	-	-	-	-
	PG	22.32 ± 1.93	-	-	-	-	-
	NS	20.20 ± 8.86	-	-	-	-	-

Note: FS is abbreviated for the fine sand group, PG for the pea gravel group, and NS for the no-substrate group. BG = between group and WG = within group. η_P_^2^ = partial eta squared (effect size: <0.01 = small, 0.01~0.06 = medium, and >0.06 = large); 95% CI = 95% confidence interval. 95% CI is presented for significant differences between FS (optimal substrate) and NS (control group); “-” indicates no significant difference (*p* > 0.05). N = 3 indicates that the replicate units are barrels, with data analyzed based on the mean values of the three compartments per barrel. * Digging and burying refers to the behavior of juveniles digging and utilizing the substrate to hide themselves; active avoidance refers to the behavior of juveniles attempting to escape the environment by swimming and scrambling to mount along the edges and corners of the barrel; and hidden rest refers to the behavior of juveniles resting concealed beneath the substrate. Identical letters indicate no significant difference; different lowercase letters indicate a significant difference (*p* < 0.05); and capital letters indicate a highly significant difference (*p* < 0.01).

**Table A2:** Effects of different substrate types on the duration of juvenile *P. cantorii* behavior (mean ± SD, N = 3).

Behavior Type [9]	Substrate Type	Duration	*F*	DF (BG, WG)	*p* Values	η_P_^2^ (Effect Size)	95% CI (FS vs. NS)
		(%)					
Bite food	FS	0.001 ± 0.001	-	-	-	-	-
	PG	0.001 ± 0.001	-	-	-	-	-
	NS	0.002 ± 0.003	-	-	-	-	-
Swallow food	FS	0.02 ± 0.02	-	-	-	-	-
	PG	0.01 ± 0.01	-	-	-	-	-
	NS	0.002 ± 0.004	-	-	-	-	-
Predation	FS	0.002 ± 0.002	-	-	-	-	-
	PG	0.001 ± 0.001	-	-	-	-	-
	NS	0.0004 ± 0.0007	-	-	-	-	-
Patrolling	FS	0.20 ± 0.07 ^A^	-	-	-	-	-
	PG	0 ^B^	34.29	2, 6	<0.001	0.924	0.12–0.28
	NS	0 ^B^	-	-	-	-	-
Extended observation	FS	0.53 ± 0.41	-	-	-	-	-
	PG	0.50 ± 0.69	-	-	-	-	-
	NS	0	-	-	-	-	-
Roaming	FS	0.57 ± 0.38 ^A^	-	-	-	-	-
	PG	0 ^B^	29.27	2, 6	<0.001	0.904	0.32–0.82
	NS	0 ^B^	-	-	-	-	-
Climbing	FS	0.03 ± 0.05	-	-	-	-	-
	PG	0	-	-	-	-	-
	NS	0	-	-	-	-	-
Digging and burying *	FS	0.46 ± 0.02 ^B^	-	-	-	-	-
	PG	0.95 ± 0.31 ^A^	18.25	2, 6	0.002	0.859	0.38–0.54
	NS	0 ^C^	-	-	-	-	-
Out of the water	FS	0.14 ± 0.02 ^A^	-	-	-	-	-
	PG	0 ^B^	75	2, 6	<0.001	0.969	0.11–0.17
	NS	0.01 ± 0.02 ^B^	-	-	-	-	-
Slow crawling	FS	2.24 ± 1.57	-	-	-	-	-
	PG	1.34 ± 0.35	-	-	-	-	-
	NS	1.09 ± 1.11	-	-	-	-	-
Going down the barrel	FS	0.20 ± 0.07 ^B^	-	-	-	-	-
	PG	0 ^C^	20	2, 6	0.002	0.87	−0.21–−0.03
	NS	0.32 ± 0.17 ^A^	-	-	-	-	-
Quick touring	FS	0.01 ± 0.01	-	-	-	-	-
	PG	0	-	-	-	-	-
	NS	0.01 ± 0.01	-	-	-	-	-
Active avoidance *	FS	1.09 ± 1.00 ^C^	-	-	-	-	-
	PG	18.79 ± 3.69 ^B^	50.26	2, 6	<0.001	0.943	−60.54–−41.44
	NS	52.08 ± 14.84 ^A^	-	-	-	-	-
Hidden rest *	FS	93.51 ± 2.28 ^A^	-	-	-	-	-
	PG	29.69 ± 25.73 ^B^	22.66	2, 6	0.001	0.883	89.85–97.17
	NS	0 ^C^	-	-	-	-	-
Lying down	FS	0.50 ± 0.64 ^C^	-	-	-	-	-
	PG	25.11 ± 10.11 ^B^	22.28	2, 6	0.001	0.881	−55.13–−36.03
	NS	46.08 ± 15.23 ^A^	-	-	-	-	-
Breathing	FS	0.51 ± 0.26 ^a^	-	-	-	-	-
	PG	0.22 ± 0.02 ^b^	5.83	2, 6	0.039	0.657	0.09–0.53
	NS	0.21 ± 0.10 ^b^	-	-	-	-	-

Note: FS is abbreviated for the fine sand group, PG for the pea gravel group, and NS for the no-substrate group. BG = between group and WG = within group. η_P_^2^ = partial eta squared (effect size: <0.01 = small, 0.01~0.06 = medium, and >0.06 = large); 95% CI = 95% confidence interval. 95% CI is presented for significant differences between FS (optimal substrate) and NS (control group); “-” indicates no significant difference (*p* > 0.05). N = 3 indicates that the replicate units are barrels, with data analyzed based on the mean values of the three compartments per barrel. * Digging and burying refers to the behavior of juveniles digging and utilizing the substrate to hide themselves; active avoidance refers to the behavior of juveniles attempting to escape the environment by swimming and scrambling to mount along the edges and corners of the barrel; and hidden rest refers to the behavior of juveniles resting concealed beneath the substrate. Identical letters indicate no significant difference; different lowercase letters indicate a significant difference (*p* < 0.05); and capital letters indicate a highly significant difference (*p* < 0.01).
